# Gene expressions associated with longer lifespan and aging exhibit similarity in mammals

**DOI:** 10.1093/nar/gkad544

**Published:** 2023-06-23

**Authors:** Masaki Takasugi, Yuya Yoshida, Yoshiki Nonaka, Naoko Ohtani

**Affiliations:** Department of Pathophysiology, Osaka Metropolitan University, Graduate School of Medicine, Osaka, Japan; Department of Pathophysiology, Osaka Metropolitan University, Graduate School of Medicine, Osaka, Japan; Department of Pathophysiology, Osaka Metropolitan University, Graduate School of Medicine, Osaka, Japan; Department of Pathophysiology, Osaka Metropolitan University, Graduate School of Medicine, Osaka, Japan

## Abstract

Although molecular features underlying aging and species maximum lifespan (MLS) have been comprehensively studied by transcriptome analyses, the actual impact of transcriptome on aging and MLS remains elusive. Here, we found that transcriptional signatures that are associated with mammalian MLS exhibited significant similarity to those of aging. Moreover, transcriptional signatures of longer MLS and aging both exhibited significant similarity to that of longer-lived mouse strains, suggesting that gene expression patterns associated with species MLS contribute to extended lifespan even within a species and that aging-related gene expression changes overall represent adaptations that extend lifespan rather than deterioration. Finally, we found evidence of co-evolution of MLS and promoter sequences of MLS-associated genes, highlighting the evolutionary contribution of specific transcription factor binding motifs such as that of E2F1 in shaping MLS-associated gene expression signature. Our results highlight the importance of focusing on adaptive aspects of aging transcriptome and demonstrate that cross-species genomics can be a powerful approach for understanding adaptive aging transcriptome.

## Graphical Abstract

**Graphical Abstract:**
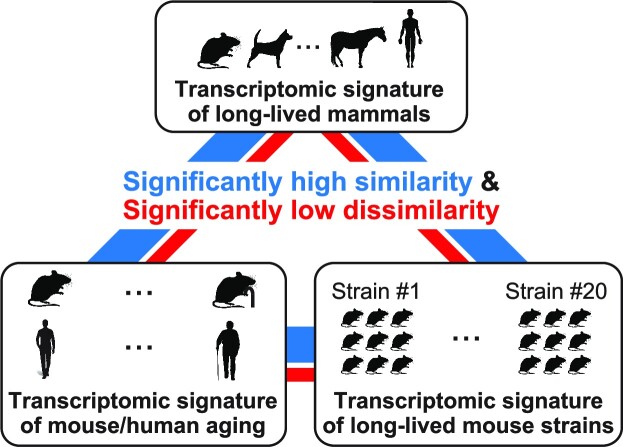

## INTRODUCTION

Owing to the recent advances in the understanding of the molecular mechanisms of aging, it is becoming clear that aging itself is a treatable condition (1). Since aging is the largest risk factor for physical functional decline and numerous diseases, development of its treatment has a potential to vastly improve the quality of life of elderly people (2). Therefore, further understanding of the mechanisms of aging is of crucial importance. In order to gain a systematic understanding of fundamental molecular processes of aging, much effort has been devoted to clarify aging-related gene expression changes (3). Microarray and sequencing technologies enabled comprehensive evaluation of aging-related changes in transcriptome (4–8), which basically represents the overall state of cells/tissues. To date, *Tabula Muris Senis* (7,8) and the Genotype-Tissue Expression (GTEx) consortium (6) provided the most comprehensive information of aging-related transcriptomic changes in mouse and human, respectively. These up-to-date large-scale analyses, together with numerous preceding studies, identified some common transcriptional features of aging, such as upregulation of inflammatory genes (3,9), which are believed to contribute to chronic inflammatory state called inflammaging. In addition to the overrepresentation of some gene ontologies, it has been discovered that the majority of aging-related gene expression changes represent reversals or extensions of developmental gene expression changes (10), suggesting that aging-related gene expression changes are not just the result of damage accumulation but are associated with the developmental program (11,12). A link between development and aging has been also suggested from a physiological point of view (12–14). Gene expression changes driven by such developmental drift and damage accumulation may contribute to aging-related deterioration (11,15). However, although aging-related transcriptomic changes have been described in great detail, their functional consequences still remain largely unproven.

Cross-species analysis is another approach to study genetic programs involved in lifespan regulation. For example, it has been reported that genes whose manipulation affects organismal lifespan are more conserved during evolution, and studies of such so-called longevity-associated genes (LAGs) have illuminated species-dependent and -independent longevity-associated pathways (16), novel protein-protein interaction networks that may contribute to longevity, and genetic links between aging and age-related diseases (17). In addition, investigation of transcriptional features of long-lived species would help to reveal pro-longevity genetic programs. Transcriptomic analysis of exceptionally long-lived species, such as the naked mole-rat (18,19), blind mole-rat (20,21), bat (22), gray whale (23) and bow-head whale (24,25) provided insights into the pathways that might potentially support their long lifespan. In a milestone study published in 2015, a group led by Vadim N Gladyshev reported a transcriptome of liver, kidney, and brain of 33 mammalian species (4 Carnovora, 2 Chiropetra, 1 Didelphimorphia, 1 Diprotodoncia, 1 Erinaceomorpha, 4 Euungulata, 1 Lagomorpha, 1 Monotremata, 8 Primates, 9 Rodentia and 1 Soricomorpha) and uncovered parallel evolution of gene expression and lifespan (26). They found that the expressions of DNA repair- and detoxification-related genes positively correlate with maximum lifespan (MLS), whereas the expressions of genes involved in central metabolism and growth hormone signaling negatively correlate with MLS. One year later the same group reported transcriptomic study of cultured skin fibroblasts of 16 mammalian species (2 Chiroptera, 1 Eulipotyphla and 13 Rodentia) and confirmed the positive correlation between MLS and the expressions of genes involved in DNA repair (27). Recently in 2022, a group led by Vera Gorbunova and Andrei Seluanov reported transcriptomic study of liver, kidney, brain, heart, lung and skin of 26 mammals (3 Eulipotyphla and 23 Rodentia) (28). While these mammals are highly diverse in their MLS, they are mostly rodents and are closely related phylogenetically. Analyses using this dataset would thus have high power to detect MLS-associated signatures, although it may be unclear whether the findings apply only to Rodentia and Eulipotyphla or also to other orders of mammals. They found that genes related to DNA repair, microtubule organization, and RNA transport are more expressed in long-lived Rodentia/Eulipotyphla while genes related to energy metabolism and inflammation are less expressed in long-lived Rodentia/ Eulipotyphla.

To date, although cross-species transcriptomic studies revealed many features of MLS-associated gene expression patterns, how expression of these genes relates to aging still remains largely unexplored. Here, we showed that gene expression patterns associated with species MLS and aging exhibited significant similarity. Moreover, transcriptional signatures of longer MLS and aging were all associated with longer lifespan in BXD (29,30) mouse strains. Our results suggest that gene expression patterns associated with species MLS contribute to extended lifespan even within a species (i.e. BXD mouse strains), and that aging-related gene expression changes overall represent adaptations that extend lifespan rather than deterioration.

## MATERIALS AND METHODS

### Identification of genes associated with MLS in mammals

Genes associated with MLS in mammals were identified as described below using amalgamated cross-species RNA-Seq dataset compiled by Fukushima and Pollock (31). This dataset includes transcriptomic data of 52 vertebrates that are normalized by TPM and corrected by surrogate variable analysis (SVA) to remove biases likely arising from heterogeneous sampling/sequencing procedures within the dataset. For the current study, we used transcriptomic data of liver, kidney, brain, heart, ovary, and testis of 29 mammals (3 Artiodactyla, 3 Carnovora, 1 Cingulata, 1 Didelphimorphia, 1 Eulipotyphla, 1 Lagomorpha, 1 Monotremata, 1 Perissodactyla, 10 Primates, 6 Rodentia and 1 Scandentia) of which their MLS is registered in AnAge database (32). Detailed information of these species, namely species name, common name, order, maximum lifespan, body weight, and age of sexual maturity are given in Supplementary Table S1. Expression data of different species were joined based on human Ensembl gene name. Expression data of non-human species that do not have 1:1 human orthologs were not joined with those of other species. For the analysis, we only used genes whose expression data were available in 10 or more species in a given tissue. A total of 16 616, 16 590, 16 175, 15 934, 14 677 and 16 001 genes passed these criteria in liver, kidney, brain, heart, ovary and testis, respectively (hereafter referred to as ‘all investigated genes’). Association between gene expression and longevity was analyzed by Spearman test or by PGLS analysis using SVA-corrected log_2_-transformed TPM values and log_2_-transformed MLS values. PGLS analysis was conducted using R libraries ‘ape" and ‘nlme’. To take phylogenetic uncertainty into account, we performed PGLS analyses on 100 phylogenetic trees downloaded from vertlife.org (33) that were generated using randomized parameters and the maximum *P*-value was used to test the association between MLS and gene expression level. Note that a set of 100 sampled trees has been suggested to capture uncertainty of parameters used for generating phylogenetic trees (33). Benjamini, Krieger and Yekutieli method (34) was used to control the FDR and a FDR of <10% was considered to be statistically significant.

### Enrichment analyses of GO terms and transcription factor targets.

Go enrichment analyses were conducted using GOrilla (35) and Enrichr (36). Enrichment of transcription factor targets was analyzed by the ChEA3 (37) or by Fisher's exact test using the Enrichr gene set library ENCODE_and_ChEA_Consensus_TFs_from_ChIP-X. All investigated genes as defined above were used as the background except for the analyses using the ChEA3 and Enrichr web tool.

### Analysis of evolutionary changes in promoter sequences of MLS-associated genes.

Sequences of 1000 bp promoter regions were retrieved using BioMart (38). The genome assembly of Damaraland mole-rat was on the Scaffold level and was not available in BioMart, and thus was excluded from the analysis. Non-redundant vertebrate JASPAR CORE motifs (39) were scanned using FIMO (40) with default settings.

### Retrieval of aging-associated genes.

The lists of genes that are differentially expressed between young and old mice tissues, and the bulk (7) and single-cell RNA-Seq data (8) of young and old mouse tissues were retrieved from the *Tabula Muris Senis* dataset. The lists of genes that are differentially expressed between young and old human tissues were retrieved from the GTEx dataset (6) via Enrichr library GTEx_Aging_Signatures_2021 (36). These signatures were generated by Evangelista et al. using limma-voom R package (41) as described in detail in their paper under the section ‘Gene expression signatures of aging from GTE’ (42). Genes differentially expressed between 20–29 years age group and either 30–39, 40–49, 50–59, 60–69, 70–79 years old group were considered to be associated with aging. Genes upregulated in some age group and downregulated in some other age group were not included in aging-associated genes. The lists of genes that are differentially expressed between 20–59 and 60–99 years old human brain were retrieved from the dataset generated by Carl W. Cotman and colleagues (4). The numbers of these aging-associated genes are provided in Supplementary Tables S2. Expression levels shown in the figures are given in TPMs or DESeq2 normalized counts (43).

### Cross-species comparison of the ratio of T cells in kidney and liver immune cells.

For mouse tissue, processed droplet scRNA-Seq data and the results of clusterization were retrieved from the *Tabula Muris Senis* dataset (8). For human tissues, count data were retrieved from the original studies (44,45) and processed using Seurat version 2.3.4 and then clustered by shared nearest neighbor method using 29 and 30 principal components for liver and kidney, respectively (resolution = 0.8). Cell clusters with high CD45 levels were extracted as immune cells and further classified by shared nearest neighbor method using 30 principal components (resolution = 0.1 for mouse kidney, 0.2 for mouse liver, 0.24 for human kidney and 0.2 for human liver). Cell clusters were then classified based on the expression levels of CD11c, CD11b, CD19 and CD3ϵ. A cell cluster in human kidney that could not be classified using the above markers was found to express high levels of kidney epithelial markers and was not considered as a part of immune cell. A cell cluster expressing high levels of HBA1 or HBA2 (red blood cell markers) were identified in mouse kidney, human kidney, and human liver, and were not considered as a part of immune cells.

### Retrieval of genes associated with longevity of rodentia/eulipothyphla and BXD strains.

The lists of genes that are correlated with MLS of Rodentia/Eulipothyphla (Spearman test, BH-adjusted *P*-value < 0.05 and |ρ| > 0.4) were retrieved from the study by Lu *et al.* (28). The lists of genes whose expression in liver and kidney correlate with the median lifespan of female mice of BXD mouse strains (|ρ| > 0.4) were retrieved from the study by Vitiello *et al.* (30). Identification of genes associated with the median lifespan of BXD mouse strains were conducted using microarray that contains one or more probes for each gene. Genes identified to be positively associated with lifespan by one probe but also identified to be negatively associated with lifespan by another probe were not included in longevity-associated genes.

### Statistical significance test of the overlap between two gene sets.

Statistical significance of the overlap between two gene sets was tested by binominal test under null hypothesis that the two gene sets overlap only by chance. All statistical tests were two-sided.

## RESULTS

### Aging-like GO enrichment of mammalian MLS-associated genes.

**Figure 1.**
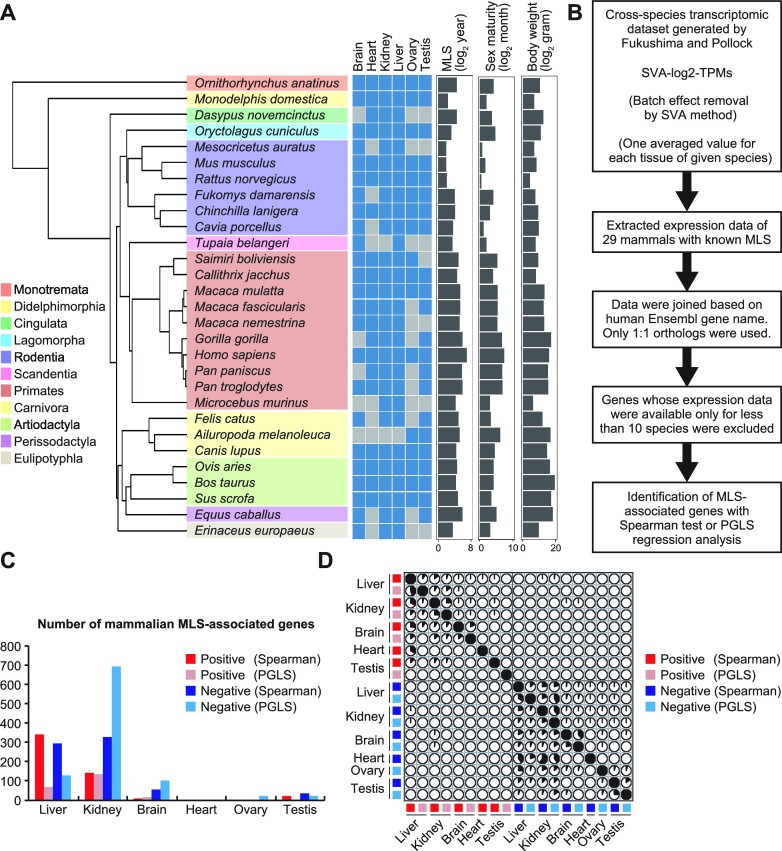
Identification of mammalian MLS-associated genes. (**A**) The names of 29 mammalian species that were used to extract mammalian MLS-associated genes and a phylogenetic tree drawn by R package ‘ape’ using phylogenetic data downloaded from VertLife.org. A heatmap shows the availability of transcriptomic data and each box is colored blue if transcriptomic data was available in the corresponding tissue of corresponding species. Right bar graphs show MLS, age of female sexual maturity, and body weight of each species. (**B**) Procedures for the identification of mammalian MLS-associated genes. (**C**) The bar graph shows the number of MLS-associated genes identified in this study. (**D**) The correlation plot represents the overlap among mammalian MLS-associated genes identified in liver, kidney, brain, heart, ovary and testis by Spearman and PGLS methods. Each pie represents the percentage of overlapping genes in the gene set shown on the left side.

In order to investigate the characteristics of MLS-associated genes, we first extracted expression data of liver, kidney, brain, heart, ovary, and testis of 29 mammals (Figure 1A, Supplementary Table S1) with known maximum lifespan (32) from amalgamated cross-species RNA-Seq dataset compiled by Fukushima and Pollock in 2020 (31). We then identified MLS-associated genes using Spearman correlation analysis as described in Figure 1B and in Materials and Methods (Supplementary Tables S2, S3). Correlation with FDR less than 10% was considered to be statistically significant. In the liver and kidney, 637 and 470 genes correlated with MLS, respectively (Figure 1C), and fewer genes correlated with age of sexual maturity or body weight (Supplementary Table S3). In brain, 68 genes correlated with MLS and about twice the number of genes correlated with age of sexual maturity and body weight. In heart, only eight genes correlated with MLS and even fewer genes correlated with age of sexual maturity or body weight. In ovary, none of the genes correlated with MLS and only one gene correlated with body weight, while as many as 127 genes correlated with age of sexual maturity. In testis, 58, 265 and 10 genes correlated with MLS, age of sexual maturity, and body weight, respectively, suggesting that cross-species differential gene expressions in reproductive organs more reflect age of sexual maturity than MLS. MLS-associated genes found in these tissues had absolute correlation coefficient values greater than 0.53 (Supplementary Table S3) and were largely tissue-specific (Figure 1D).

We next examined the enrichment of gene ontology (GO) terms relative to all investigated genes, and found that genes whose expression in the kidney was positively and negatively correlated with mammalian MLS were enriched with inflammatory and mitochondrial genes, respectively (Supplementary Figure S1). Considering that high expression of inflammatory genes and low expression of mitochondrial genes are primary features of aging (3,9), these results may suggest some similarity between MLS-associated genes and aging-associate genes.

### MLS- and mouse aging-associated genes overlap significantly.

Although our MLS-associated genes exhibited enrichment of GO terms that is reminiscent of aging transcriptome, it is not clear at this point whether it is just that different genes within the same GO term is associated with mammalian MLS and aging, or whether MLS-associated genes significantly overlap with aging-associated genes (i.e. genes whose expression was correlated with aging). To address this question, we compared MLS-associated genes with the genes whose expression changes during aging in mouse liver, kidney, brain and heart, which were identified by Wyss-Coray and Quake labs and *Tabula Muris* consortium (7) (Supplementary Table S2, S4).

**Figure 2.**
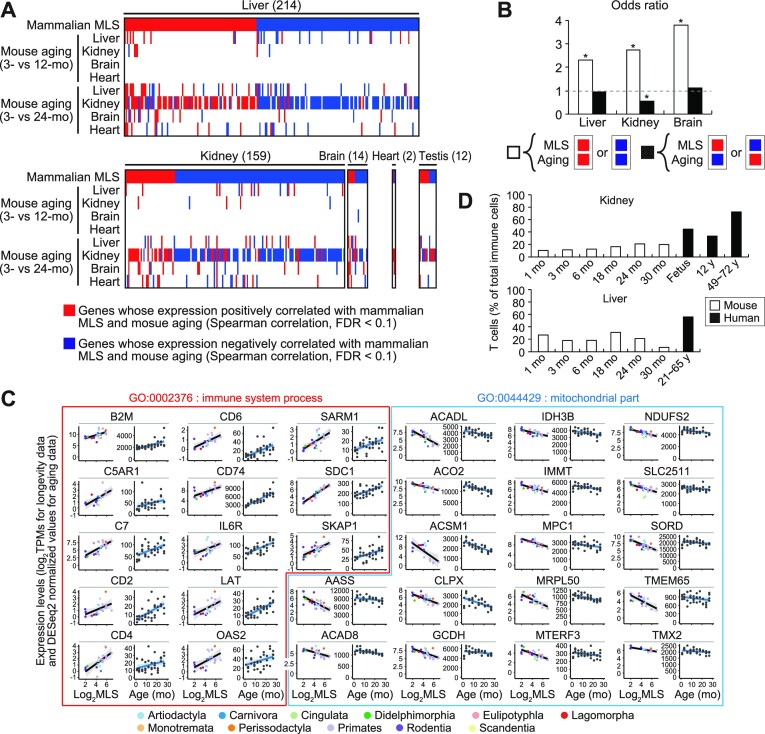
A significant similarity between gene expression patterns associated with mammalian MLS and mouse aging. (**A**) The heatmaps show the overlap between mouse aging-associated genes and mammalian MLS-associated genes identified by Spearman test (FDR < 0.1). Each column represents a single gene and the total number of genes shown in the heatmap is indicated in the parenthesis. The top rows show whether the gene was positively (red) or negatively (blue) associated with mammalian MLS in the tissue shown on the top of the heatmap. Other rows indicate whether the gene was upregulated (red), downregulated (blue), or unchanged (white) during aging in mouse in the tissue shown on the left of the heatmap. Only MLS-associated genes whose expression was affected by aging in either of the tissues are shown in the heatmaps. (**B**) The bar graphs represent the odds ratios of concordant (white bars) and discordant genes (black bars) (see main text for the definition). The odds ratios of concordant and discordant genes represent the concordance and discordance, respectively, between transcriptional signatures of mouse aging and longer MLS identified by Spearman test. (**C**) The plots show the expression levels of indicated genes in the kidney of different species or in male mice of different ages. The plots surrounded by red lines show the expression levels of ‘immune system process’ (GO:0002376) genes that were positively correlated with both mammalian MLS and mouse aging. The plots surrounded by blue lines show the expression levels of ‘mitochondrial part’ (GO:0044429) genes that were negatively correlated with both mammalian MLS and mouse aging. (**D**) The bar graphs show the percentages of T cells in the total immune cells in kidney and liver. Cell types were determined based on scRNA-seq data. The white and black bars show the percentages of T cells in mouse and human, respectively. * FDR < 0.05 (binominal test).

Surprisingly, we found a significant overlap between MLS- and aging-associated genes in liver, kidney, and brain (Figure 2A). That is, genes positively correlated with both MLS and aging, and genes negatively correlated with both MLS and aging, which we hereafter collectively refer to as ‘concordant genes’, appeared significantly more often than expected under the assumption of independence of MLS- and aging-associated genes (Figure 2B). In addition, in the kidney, genes positively correlated with MLS and simultaneously negatively correlated with aging, and genes negatively correlated with MLS and simultaneously positively correlated with aging, which we hereafter collectively refer to as ‘discordant genes’, appeared significantly less often than expected under the assumption of independence of MLS- and aging-associated genes (Figure 2B). This indicates that the discordance between MLS- and aging-associated gene expression patterns were smaller than expected by chance. These results collectively support the similarity between the transcriptional signatures of aging and longer MLS. This similarity was maintained in the kidney even after removing genes correlated with age of sexual maturity or body weight (Supplementary Figure S2). Thus, although these factors are known to be associated with species MLS, they are not the primary determinant of the similarity between the transcriptional signatures of aging and longer MLS.

Genes positively correlated with both mammalian MLS and mouse aging in the kidney were enriched with inflammation-related genes (Supplementary Figure S1). These inflammatory genes included several T cell markers, such as CD2, CD4 and CD6, suggesting that the enrichment of inflammation-related genes at least partially reflects an increase in T cells in longer-lived mammals (Figure 2C). Indeed, re-analysis of *Tabula Muris Senis* (8) and public human scRNA-Seq datasets (44,45) showed that the percentages of T cells in immune cells in kidney and liver were higher in human than in mouse (Figure 2D). Genes negatively correlated with both mammalian MLS and mouse aging in the kidney were enriched with mitochondrial genes (Figure 2C, Supplementary Figure S1). Re-analysis of *Tabula Muris Senis* scRNA-Seq dataset (8) showed that these mitochondrial genes tend to be downregulated during aging in kidney parenchymal cells, such as proximal convoluted tubule epithelial cells (Supplementary Figure S3).

Next, in order to corroborate the similarity between MLS- and aging-associated genes, we extracted MLS-associated genes using another method, namely phylogenetic generalized least squares (PGLS) regression analysis (46) (Supplementary Table S2, S3). Unlike Spearman test, PGLS is a parametric test but does take the phylogenetic non-independence of species into consideration. As a result, we found 201, 832, and 114 MLS-associated genes in liver, kidney, and brain, respectively (FDR < 0.1). Ovary and testis had only small numbers of MLS-associated genes (23 and 22 genes, respectively). No MLS-associated gene was identified in the heart (Supplementary Table S2, S3). MLS-associated genes identified by PGLS method were largely non-overlapping with those identified by Spearman method (Figure 1D). The discrepancy is likely due at least partially to the limited sensitivity of statistical analyses but it should be noted that the overlap between the lists of MLS-associated genes identified by these methods was still statistically significant (Supplementary Figure S4A). Nonetheless, MLS-associated gene expression patterns identified by PGLS method also exhibited significant similarity with gene expression patterns associated with mouse aging (Supplementary Figure S4B and C, Tables S2 and S4). The fact that Spearman and PGLS methods pointed to the same conclusion indicates its robustness. In order to enhance the comprehensiveness of the following analyses, we hereafter combined the lists of MLS-associated genes obtained by these two methods.

**Figure 3.**
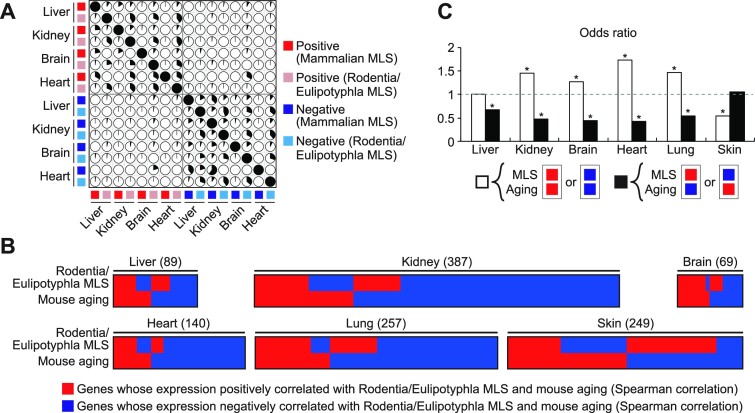
A significant similarity between gene expression patterns associated with Rodentia/Eulipotyphla MLS and mouse aging. (**A**) The correlation plot represents the overlap among mammalian and Rodentia/Eulipotyphla MLS-associated genes identified in liver, kidney, brain, and heart. Each pie represents the percentage of overlapping genes in the gene set shown on the left side. (**B**) The heatmaps show the overlap between genes associated with Rodentia/Eulipotyphla MLS and mouse aging. Each column represents a single gene and the total number of genes shown in the heatmap is indicated in the parenthesis. The top rows show whether the gene was positively (red) or negatively (blue) associated with Rodentia/Eulipotyphla MLS in the tissue shown on the top of the heatmap. The bottom rows indicate whether the gene was upregulated (red) or downregulated (blue) during aging in mouse in the tissue shown on the top of the heatmap. Only MLS-associated genes whose expression was also affected by aging are shown in the heatmaps. (**C**) Bar graphs represent the odds ratios of concordant (white bars) and discordant genes (black bars). * FDR < 0.05 (binominal test).

In order to further verify our finding, we next investigated the similarity between MLS- and aging-associated gene expression signatures using MLS-associated genes of liver, kidney, brain, heart, lung, and skin of Rodentia and Eulipotyphla that have been identified by Lu *et al.* (28). Since 23 out of 26 mammals used in their study were rodents, species-dependent gene expression would be less affected by mammalian order-dependent traits irrelevant to MLS. Rodentia/Eulipotyphla MLS-associated genes identified by Lu *et al.* were largely non-overlapping with mammalian MLS-associated genes identified by us (Figure 3A). Nonetheless, Rodentia/Eulipotyphla MLS-associated gene expression patterns exhibited significant similarity with gene expression patterns associated with mouse aging in liver, kidney, brain, heart, and lung (Figure 3B and C, Supplementary Table S5, S6). Genes correlated with mouse skin aging was the exception and their overlap with MLS-associated genes was significantly smaller than expected by chance. This could be explained by our observation that aging-related gene expression changes were highly fluctuating in mouse skin (Supplementary Figure S5). Thus, unlike other tissues, aging-related genes in mouse skin are less likely to represent cumulative effects of aging, which could be due to the fact that external factors play major roles in skin aging.

### MLS- and human aging-associated genes overlap significantly.

**Figure 4.**
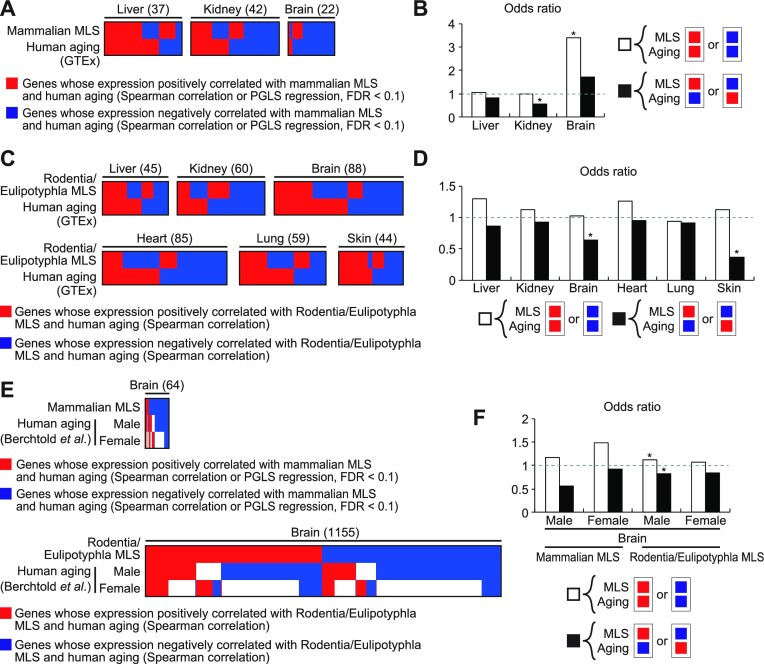
A significant similarity between gene expression patterns associated with species MLS and human aging. (A, C, E) The heatmaps show the overlap between genes associated with species MLS and human aging. Each column represents a single gene and the total number of genes shown in the heatmap is indicated in the parenthesis. The top rows show whether the gene was positively (red) or negatively (blue) associated with species MLS in the tissue shown on the top of the heatmap. The bottom rows indicate whether the gene was upregulated (red), downregulated (blue), or unchanged (white) during aging in human in the tissue shown on the top of the heatmap. Only MLS-associated genes whose expression was also affected by aging are shown in the heatmaps. (**A**) The overlap between genes associated with mammalian MLS and human aging (according to GTEx database). (**C**) The overlap between genes associated with Rodentia/Eulipotyphla MLS and human aging (according to GTEx database). (**E**) The overlap between genes associated with species MLS and human brain aging (according to the study by Berchtold *et al*). (**B**, **D**, **F**) The bar graphs shown in (B), (D) and (F) represent the odds ratios of concordant (white bars) and discordant genes (black bars) in the genes shown in (A), (C) and (E), respectively. * FDR < 0.05 (binominal test).

Although we established the similarity between gene expression patterns associated with mouse aging and the MLS of Rodentia/Eulipotyphla or a wider range of mammals (hereafter collectively refer to as species MLS), it remains to be tested whether aging-associated gene expression patterns of other species, most importantly human, also exhibit similarity to MLS-associated gene expression patterns. We therefore retrieved genes that are associated with human aging from the GTEx dataset (6) via Enrichr library (36) and tested their relationship with MLS-associated genes (Supplementary Table S2 and S6). Odds ratios of concordant genes were higher than 1 in mammalian MLS-associated genes in two out of three tissues (liver and brain) and in Rodentia/Eulipotyphla MLS-associated genes in five out of six tissues (liver, kidney, brain, heart, and skin) (Figure 4A–D). On the other hand, odds ratios of discordant genes were lower than 1 in mammalian MLS-associated genes in two out of three tissues (liver and kidney) and in Rodentia/Eulipotyphla MLS-associated genes in all six tissues. Importantly, odds ratios of concordant genes were higher than those of discordant genes in all cases. The similarity between species MLS- and human aging-associated gene expression patterns was statistically significant in kidney, brain, and skin (Figure 4B and D). In order to corroborate this finding, we retrieved genes associated with human brain aging from another independent dataset generated by Carl W. Cotman and colleagues (4). Using this dataset, we confirmed that gene expression pattern associated with MLS in the brain exhibited a significant similarity to gene expression pattern of human brain aging (Figure 4E and F). Taken together, our results indicate the similarity between the transcriptional signatures of longer MLS and aging of mouse and human. Genes positively and negatively correlated with species MLS both contributed to the similarity between MLS- and aging-associated gene expression patterns (Supplementary Figure S6).

### MLS- and aging-associated expressions are both linked to longer lifespan.

**Figure 5.**
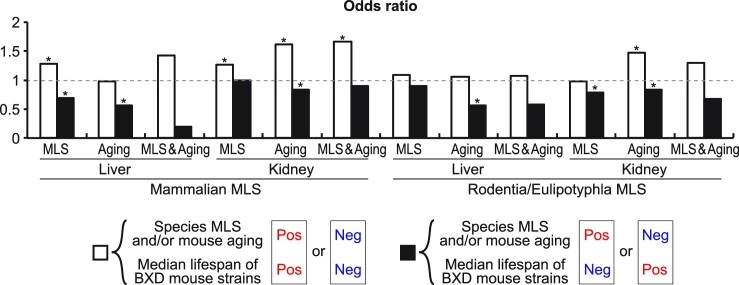
Gene expression patterns associated with species MLS and/or mouse aging exhibit significant similarities to those associated with the lifespan of genetically heterogeneous mouse strains. The white bars indicated as ‘MLS’ show the odds ratios of genes positively correlated with both mouse lifespan and species MLS, and genes negatively correlated with both mouse lifespan and species MLS. The white bars indicated as ‘Aging’ show the odds ratios of genes positively correlated with both mouse lifespan and mouse aging, and genes negatively correlated with both mouse lifespan and mouse aging. The white bars indicated as ‘MLS & Aging’ show the odds ratios of genes positively correlated with all mouse lifespan, species MLS, and mouse aging, and genes negatively correlated with all mouse lifespan, species MLS, and mouse aging. The black bars indicated as ‘MLS’ show the odds ratio of genes positively correlated with mouse lifespan and simultaneously negatively correlated with MLS, and genes negatively correlated with mouse lifespan and simultaneously positively correlated with MLS. The black bars indicated as ‘Aging’ show the odds ratio of genes positively correlated with mouse lifespan and simultaneously negatively correlated with mouse aging, and genes negatively correlated with mouse lifespan and simultaneously positively correlated with mouse aging. The black bars indicated as ‘MLS & Aging’ show the odds ratios of genes positively correlated with mouse lifespan and simultaneously negatively correlated with both species MLS and mouse aging, and genes negatively correlated with mouse lifespan and simultaneously positively correlated with both species MLS and mouse aging. * FDR < 0.05 (binominal test).

Similarity between gene expression patterns associated with MLS and aging might suggest that a significant portion of aging-related gene expression changes is actually beneficial rather than harmful for longevity. In order to investigate how MLS- and aging-associated genes relate to the variation in lifespan within a mammalian species, we next compared MLS- and aging-associated gene expression patterns with those associated with longer median lifespan in genetically heterogeneous BXD mouse strains (29,30) that were generated by crossing C57BL/6J and DBA/2J mice. Gene expression patterns associated with longer median lifespan in these strains were identified in liver and kidney (and also in eye, bone and adrenal glands) in a previous study by Ewald *et al.* (30). In liver and kidney, genes positively correlated with both species MLS and mouse lifespan, and genes negatively correlated with both species MLS and mouse lifespan, appeared significantly more often than expected by chance. On the other hand, genes positively correlated with species MLS and simultaneously negatively correlated with mouse lifespan, and genes negatively correlated with species MLS and simultaneously positively correlated with mouse lifespan, appeared significantly less often than expected by chance (Figure 5). These results suggest that gene expression patterns associated with species MLS contribute to extended longevity even within a species. Importantly, the same significant similarities were observed between gene expression patterns associated with mouse aging and mouse lifespan. Gene expressions commonly associated with species MLS and mouse aging also exhibited similarity to those associated with mouse lifespan (Figure 5, Supplementary Figure S7). These results suggest that aging-related gene expression changes overall represent adaptations that extend lifespan rather than deterioration.

**Figure 6.**
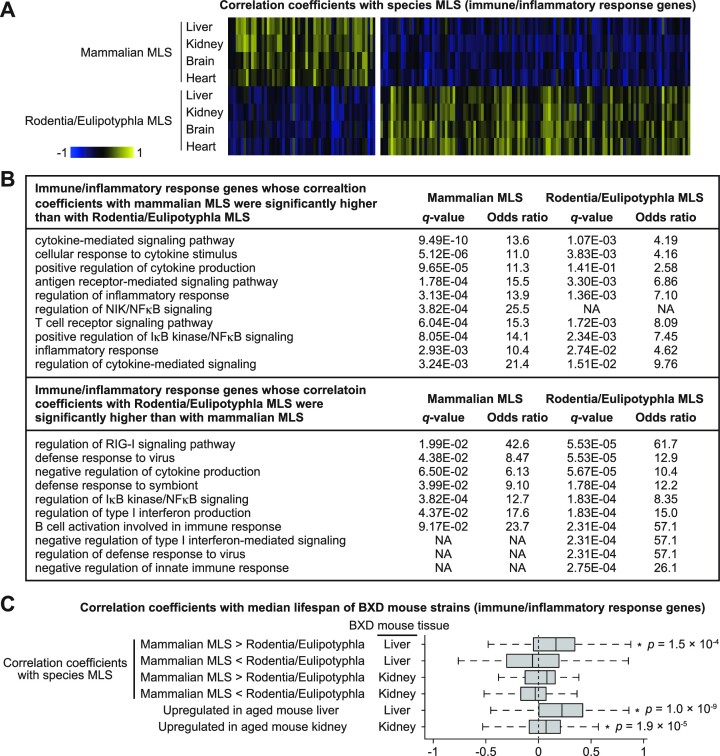
Immune/inflammatory response genes are differentially associated with MLS of Rodentia/Eulipotyphla and those of a wider range of mammals. (**A**) The heatmaps show the Spearman coefficients of correlation between species MLS and gene expressions. Each column represents a Spearman coefficient of single gene. Only immune/inflammatory genes (GO:0006945 and GO:0006955) whose Spearman coefficients were significantly different (two-tailed *t* test, FDR < 0.1) between tissues of Rodentia/Eulipotyphla and those of a wider range of mammals are shown in the figure. (**B**) The top 10 enriched GO biological process terms of the genes shown in (A). (**C**) The boxplots show the coefficients of correlation between median lifespan of BXD mouse strains and gene expression. Only immune/inflammatory genes (GO:0006945 and GO:0006955) that were upregulated during aging in mouse liver or kidney, or those whose Spearman coefficients were significantly different (two-tailed *t* test, FDR < 0.1) between tissues of Rodentia/Eulipotyphla and those of a wider range of mammals are shown in the figure. * *P*-value < 0.05 (one-sample Wilcoxon test).

Although both mammalian and Rodentia/Eulipotyphla MLS-associated genes strongly supported our conclusion, it should be noted that these two gene sets have very different relationships with immune regulation. That is, while we found that inflammatory genes were overrepresented in genes that are positively correlated with mammalian MLS, Lu *et al.* (28) reported such overrepresentation in genes that are negatively correlated with Rodentia/Eulipotyphla MLS. To better understand this difference, we extracted immune/inflammatory response genes whose Spearman coefficients of correlation between species MLS and expressions in liver, kidney, brain, and heart were significantly different between mammals and Rodentia/Eulipotyphla (Figure 6A). We found that genes involved in positive regulation of cytokine production and NFκB signaling tend to be positively and negatively correlated with mammalian and Rodentia/Eulipotyphla MLS, respectively. On the other hand, genes involved in negative regulation of cytokine production and NFκB signaling tend to be negatively and positively correlated with mammalian and Rodentia/Eulipotyphla MLS, respectively (Figure 6B). Importantly, immune/inflammatory response genes whose correlation coefficients with mammalian MLS were significantly higher than with Rodentia/Eulipotyphla MLS showed a weak but significant positive correlation with median lifespan of BXD mouse strains (Figure 6C). Immune/inflammatory response genes that were upregulated during aging in mouse liver and kidney were also positively correlated with mouse lifespan. On the other hand, immune/inflammatory response genes whose correlation coefficients with Rodentia/Eulipotyphla MLS were significantly higher than with mammalian MLS did not correlated with mouse lifespan. Thus, adaptation of immune regulation to longer lifespan evolved in different ways in Rodentia/Eulipotyphla and other mammals.

**Figure 7.**
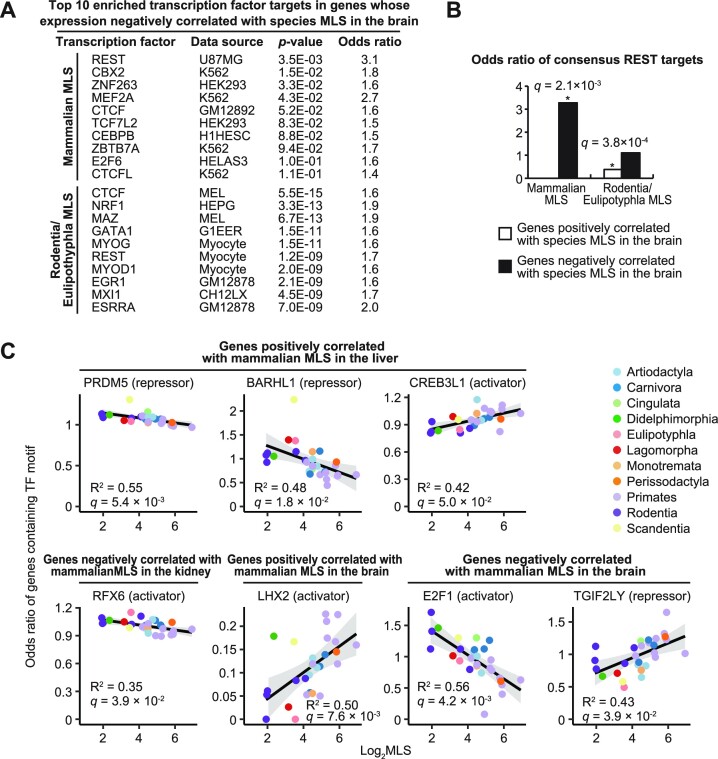
TFs regulating MLS-associated genes expression and their evolutionary changes. (**A**) Top 10 transcription factor targets that were overrepresented in the genes whose expression in the brain negatively correlated with species MLS. (**B**) The bar graph shows the odds ratios of consensus REST target genes in the genes whose expression in the brain correlated with species MLS. (**C**) The plots show the odds ratio of genes containing at least one motif of the indicated TFs in their 1000 bp promoter regions. * FDR < 0.05 [Fisher's exact test for (A, B), and Spearman correlation test for (C)].

Our finding that gene expressions associated with aging and longer lifespan exhibit similarity aligns well with a previous argument by de Magalhães *et al.* (9) that many of aging-related gene expression changes could represent adaptations to aging. Aging-related increase in the expression of transcriptional repressor REST (RE1-Silencing Transcription Factor) is a proven example of such adaptations. In human brain, age-related upregulation of REST is beneficial for longevity and cognitive performance (47,48). Therefore, we tested whether such adaptation also occurred during the evolution of longer lifespan. Enrichment analysis of transcription factor (TF) targets using ChEA3 (37) and ENCODE ChIP-seq library showed that REST targets were the most enriched TF targets in genes whose expression in the brain negatively correlated with mammalian MLS (Figure 7A). In genes whose expression in the brain negatively correlated with Rodentia/Eulipotyphla MLS, the top 6th enriched TF targets were those of REST. Moreover, consensus REST targets (i.e. genes that were commonly identified to be bound by REST in multiple experiments) were significantly over- and underrepresented in the genes whose expression in the brain was negatively and positively correlated with species MLS, respectively (Figure 7B). Thus, suppression of REST targets that is protective against aging-related brain dysfunction seems to occur both during aging and the evolution of longer lifespan.

### TFs regulating the evolutionary changes in the expression of MLS-associated genes.

It is important to note that the aforementioned results do not necessarily indicate that REST activity itself correlates with MLS in the brain. Rather, REST binding sites might have been increased selectively in the promoter regions of genes associated with MLS in the brain. It is also very possible that the evolutionary changes in the expression of these REST- and MLS-associated genes were driven by other TFs. Understanding of the regulatory mechanisms that had driven the evolutionary changes in the expression of MLS-associated genes is of great importance, since the effects of MLS-associated gene expression on longevity could depend on the biological context. In order to investigate such regulatory mechanisms, we analyzed the evolutionary changes in the presence of TF binding sequences in the 1000 bp upstream regions of transcriptional start sites (TSS) of mammalian MLS-associated genes using FIMO (40) and vertebrate JASPAR CORE position weight matrices (39). We limited our TFBS analysis to 1000 bp upstream regions of TSS due to technical reasons, although it should be kept in mind that other regions also contain many TFBS. Rodentia/Eulipotyphla MLS-associated genes were not investigated because promoter sequences were not available for the majority of species used in the comparative study (28). For each motif and each species, we calculated (1) the ratio of genes containing at least one motif in their promoters and (2) the average number of motifs per promoter for MLS-associated genes and background genes (i.e. all investigated genes). We then calculated the odds ratios of these two values between MLS-associated genes and background genes and tested their correlation with MLS (Spearman test). Motif was considered to be associated with MLS genes if the odds ratios of both of these values were significantly correlated with MLS (FDR < 5%). As a result, we found seven TF binding motifs whose presence in the promoter regions of mammalian MLS-associated genes correlated with MLS (Figure 7C, Supplementary Table S7), suggesting that MLS evolved in parallel with promoter sequences of MLS-associated genes. Importantly, evolutionary changes in the presence of these transcriptional activator- and repressor-binding motifs were all consistent with the evolutionary changes in the expression of MLS-associated genes (Figure 7C). For example, in genes whose expression in the brain positively correlated with MLS, the presence of the binding motif of transcriptional activator LHX2, which has been reported to play major roles in forebrain development (49), was positively correlated with MLS. On the other hand, in genes whose expression in the brain negatively correlated with MLS, the presence of the binding motifs of transcriptional activator E2F1 (50) and putative transcriptional repressor TGIF2LY (51) was negatively and positively correlated with MLS, respectively. Notably, genes whose expression in the brain negatively correlated with MLS not only lost binding motifs of an activator E2F (E2F1) during the evolution of longevity (Figure 7C), but were also enriched with genes targeted by a repressor E2F (E2F6) (50) in the longest-lived primate (human) (Figure 7A). These consistent results suggest that E2F transcription factors rather than REST had shaped MLS-associated gene expression signature in the brain during the evolution of longevity.

## DISCUSSION

**Figure 8.**
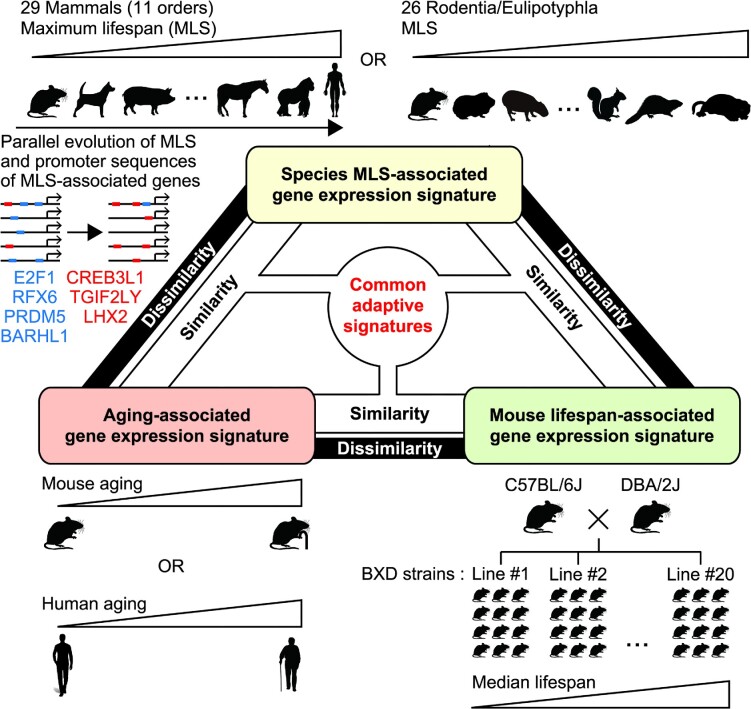
A summary of the study.

Our conclusions are based on the analyses of two different types of MLS-associated genes, namely those identified by Lu *et al.* (28) in Rodentia/Eulipotyphla and those identified by us in a wider range of mammals. Identification of MLS-associated genes in Rodentia/Eulipotyphla would be less affected by mammalian order-dependent traits irrelevant to MLS. On the other hand, findings obtained from the investigations of genes associated with longevity in a wider range of mammals including primates and human would more likely to be relevant to human biology. Importantly, MLS-associated genes identified using Spearman and PGLS methods both lead to the same conclusions. The complementarity of these analyses therefore enhances the robustness and generality of our conclusions (Figure 8). Regarding the identification of MLS-associated genes, two novel sophisticated methods using ranking-based method (23) and machine learning analysis (52) have been recently developed by groups led by Alexey A. Moskalev, Vadim E. Fraifeld and Robi Tacutu, and their utilization may allow identification of additional overlaps between MLS- and aging-associated genes.

In this study, we clearly showed that genes correlated with mouse/human aging and species MLS overlap significantly. MLS-associated gene expression signature is likely contributing to longer lifespan and thus our results suggest that a significant portion of aging-related gene expression changes could be beneficial for longevity. Although dysregulated inflammatory responses underlie pathogenesis of many diseases, their physiological functions are also crucial for tumor suppression (53). High expression of inflammatory genes in long-lived mammals may represent better immunity. Indeed, we found that gene expression patterns associated with species MLS and aging were all associated with longer lifespan in genetically heterogeneous mouse strains. This strongly supports the notion that gene expression patterns associated with species MLS contribute to extended lifespan even within a species, and that aging-related gene expression changes overall represent adaptations that extend lifespan.

There are some caveats regarding the interpretation of our results. First, considering that loss of proteostasis is a major hallmark of aging (54), age-related changes in mRNA levels may tend not to be reflected in protein levels. In the current study, we only analyzed mRNA levels and it is not clear if the same conclusion could be drawn from the same analysis using the levels of functional proteins instead of mRNA levels. Second, expression data used in this study was quantified at the gene level and not at the transcript level. Thus, we cannot exclude the possibility that age- or species-dependent splicing has some influence on our results. Third, changes in mRNA levels during aging and in the evolution of longevity may be the result rather than the cause of aging/longevity. For example, age-related decreases in mRNA levels may reflect DNA damage blocking their transcription, and longer-lived species may downregulate damage-vulnerable gene transcripts. Fourth, molecular changes observed during aging are also often observed during development (10,11,55–57). Many development-associated genes are subsumed within aging-associated genes, especially when the latter were identified by comparing young adult and old animals. Therefore, the similarity between gene expressions associated with aging and longer lifespan may be at least partially due to a link between genetic programs regulating development and longevity. Finally, it should be noted that although genes associated with aging and longer lifespan exhibited statistically significant similarity, these two gene sets were still largely non-overlapping with each other. One of the major reasons for this is that genes identified to be associated with aging or longer lifespan highly depend on species, strains, sex, conditions of animals, and methods used for the analysis. For example, as described above for Spearman and PGLS methods, each statistical method has strengths and weaknesses. In this regard it should be also noted that DESeq2 (43) that was used to identify mouse aging-related genes has been shown to severely understate FDR values when the sample size is large (58). Also, it has been reported that genes associated with aging greatly vary among 129sv, BALB/c, CBA, DBA, C57BL/6, C3H and B6C3F mice (59). If the majority of genes associated with longer lifespan in a given set of species overlapped with genes associated with aging in a given strain of animal, these MLS-associated genes would not overlap to the same extent with genes associated with aging in other strains or species. Therefore, if genes associated with longer lifespan and aging have similarity in mammals regardless of species, strains, sex, and other conditions, the overlap between these two gene sets, even if statistically significant, theoretically has to be weak.

## Supplementary Material

gkad544_Supplemental_Files

## Data Availability

All the data analyzed in this study were retrieved from freely accessible public databases as described above. Source data underlying the figures are provided in the supplementary tables.
