# Supplementary material for: Gene expressions associated with longer lifespan and aging exhibit similarity in mammals
Source: Nucleic Acids Res. 2023 Jun 23;51(14):7205–19. doi: 10.1093/nar/gkad544 (PMC10415134; doi:10.1093/nar/gkad544)
Supplement: gkad544_Supplemental_Files [file gkad544_supplemental_files.zip › Supplementary_Information.pdf]

# Gene expressions associated with longer lifespan and aging exhibit similarity in mammals

Masaki Takasugi*, Yuya Yoshida, Yoshiki Nonaka, Naoko Ohtani*

*Correspondence should be addressed to

M.T. (masaki.takasugi@omu.ac.jp) and N.O. (naoko.ohtani@omu.ac.jp)

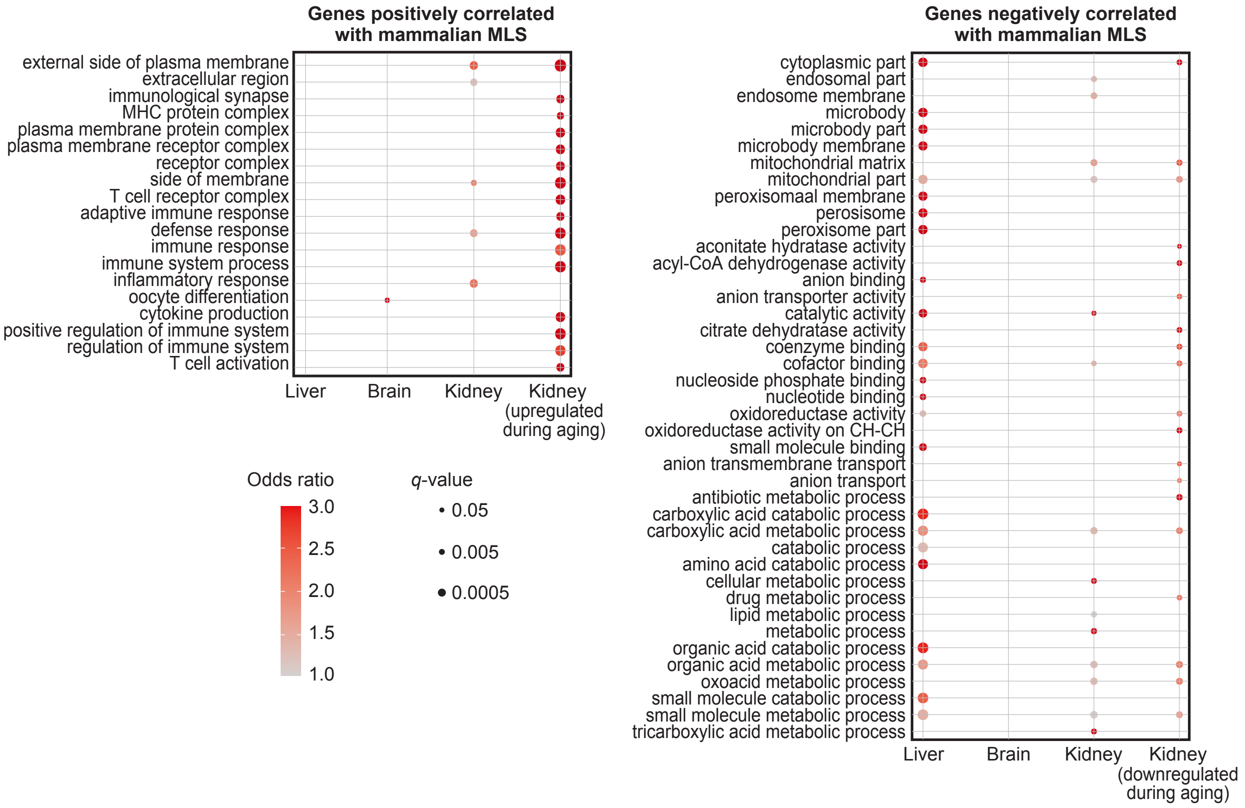


**Figure S1. GO enrichment analysis of mammalian MLS-associated genes.** The plots represent the overrepresentation of GO terms in mammalian MLS-associated genes. GO enrichment was analyzed using GOrilla and *q*-value of less than 0.05 was considered to be statistically significant. All investigated genes, namely the genes whose expression data were available in 10 or more species in a given tissue, were used as the background gene list. Up to the top 8 significantly enriched GO biological process, molecular function, and cellular component terms are shown in the figure. For the kidney, the enrichment analysis was also performed for the genes that are positively correlated with both mammalian MLS and mouse aging, and the genes that are negatively correlated with both mammalian MLS and mouse aging.

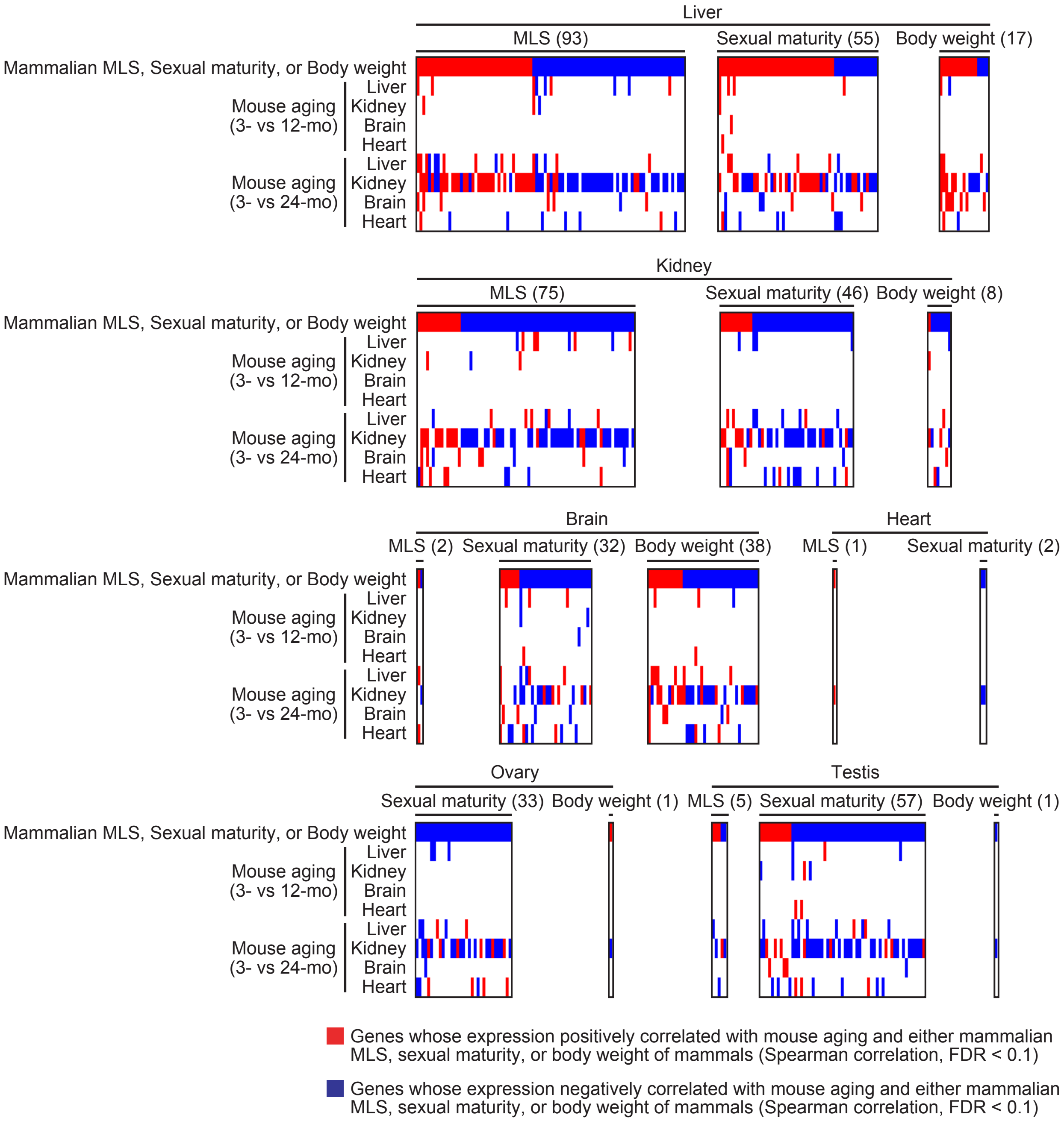


**Figure S2. Investigation of genes that were specifically correlated with MLS, age of sexual maturity, or body weight.** The heatmaps show the overlap between mouse aging-associated genes and genes identified by Spearman test to be significantly correlated only with MLS, age of female sexual maturity, or body weight. Each column represents a single gene and the total number of genes shown in the heatmap is indicated in the parenthesis. The top rows show whether the gene was positively (red) or negatively (blue) coorrelated with mammalian MLS in the tissue shown on the top of the heatmap. Other rows indicate whether the gene was upregulated (red), downregulated (blue), or unchanged (white) during aging in mouse in the tissue shown on the left of the heatmap. Only MLS-associated genes whose expression was affected by aging in either of the tissues are shown in the heatmaps.

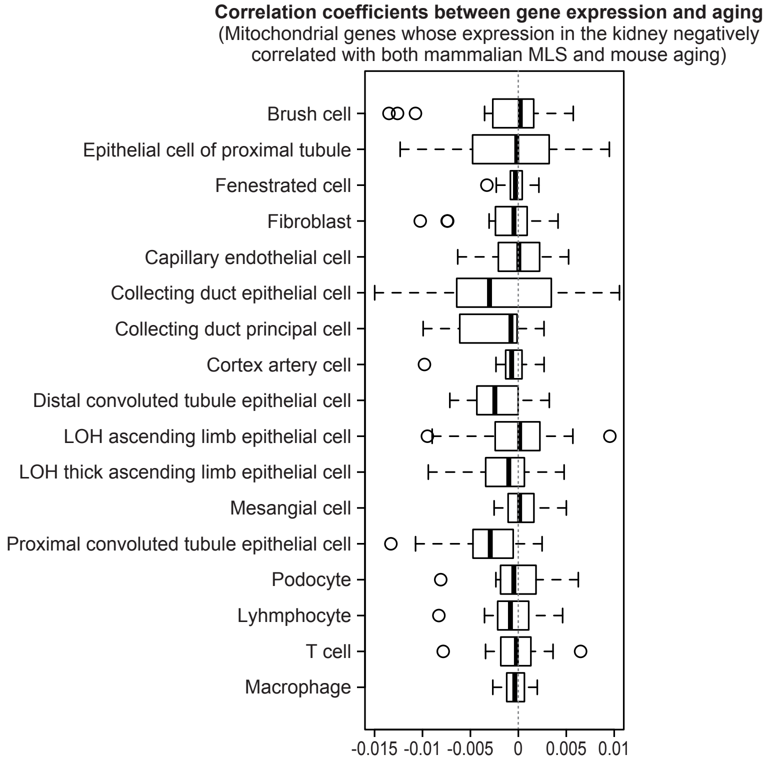


**Figure S3. Investigation of cell types responsible for MLS- and aging-associated expression of mitochondrial genes in the kidney.** The plot shows the age-coefficients of the expressions of mitochondrial genes (GO:0044429) in mouse kidney. Data are shown only for genes that were negatively correlated with both mammalian MLS and mouse kidney aging (according to *Tabula Muris Senis* bulk RNA-Seq data). These age-coefficients, which were retrieved from the study by the *Tabula Muris* consortium, were calculated based on scRNA-Seq data and are shown for all cell types identified in the kidney.

**A**

| | Spearman | PGLS | Overlap | Odds ratio | *p*-value |
|---|---|---|---|---|---|
| Liver | 637 | 201 | 74 | 9.6 | $4.2 \times 10^{-53}$ |
| Kidney | 470 | 832 | 165 | 7.0 | $4.5 \times 10^{-97}$ |
| Brain | 68 | 114 | 24 | 50.1 | $2.0 \times 10^{-35}$ |

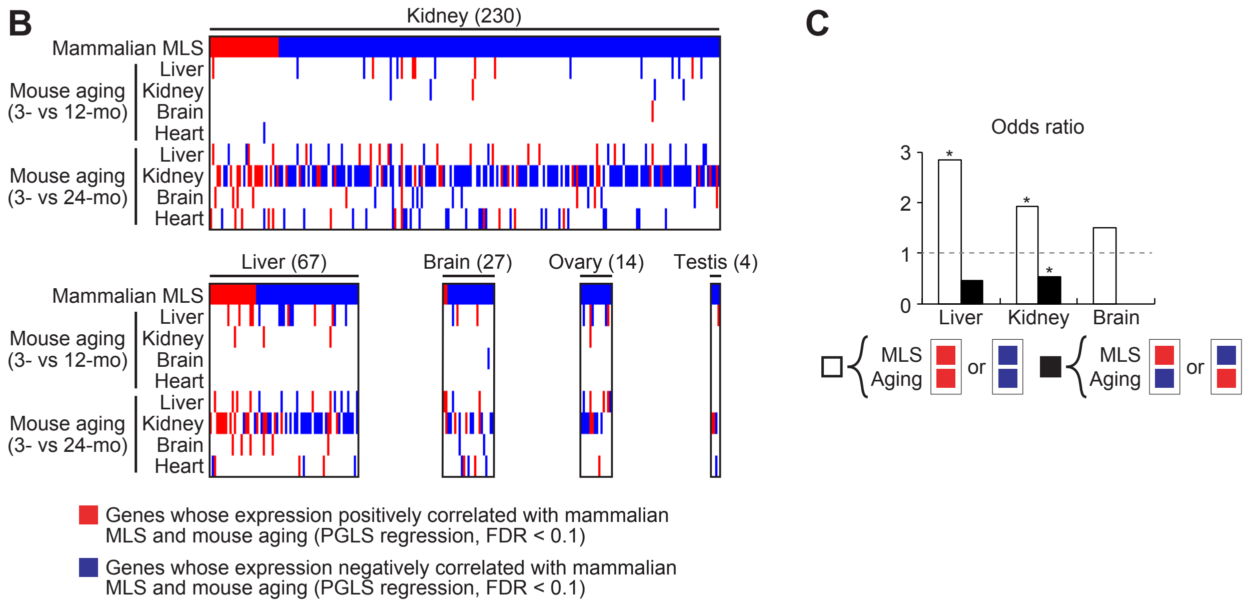


**Figure S4. PGLS regression analysis also supports the significant similarity between gene expression patterns associated with mammalian MLS and mouse aging.** (**A**) Statistical significance of the overlap between MLS-associated genes identified by Spearman and PGLS methods were tested using Fisher's exact test. (**B**) The heatmaps show the overlap between mouse aging-associated genes and mammalian MLS-associated genes identified by PGLS regression analysis (FDR < 0.1). Each column represents a single gene and the total number of genes shown in the heatmap is indicated in the parenthesis. The top rows show whether the gene was positively (red) or negatively (blue) associated with mammalian MLS in the tissue shown on the top of the heatmap. Other rows indicate whether the gene was upregulated (red), downregulated (blue), or unchanged (white) during aging in mouse in the tissue shown on the left of the heatmap. Only MLS-associated genes whose expression was affected by aging in either of the tissues are shown in the heatmaps. (**C**) The bar graphs represent the odds ratios of concordant (white bars) and discordant genes (black bars) (see main text for the definition). The odds ratios of concordant and discordant genes represent the concordance and discordance, respectively, between transcriptional signatures of mouse aging and longer MLS identified by PGLS regression analysis. * FDR < 0.05 (binominal test).

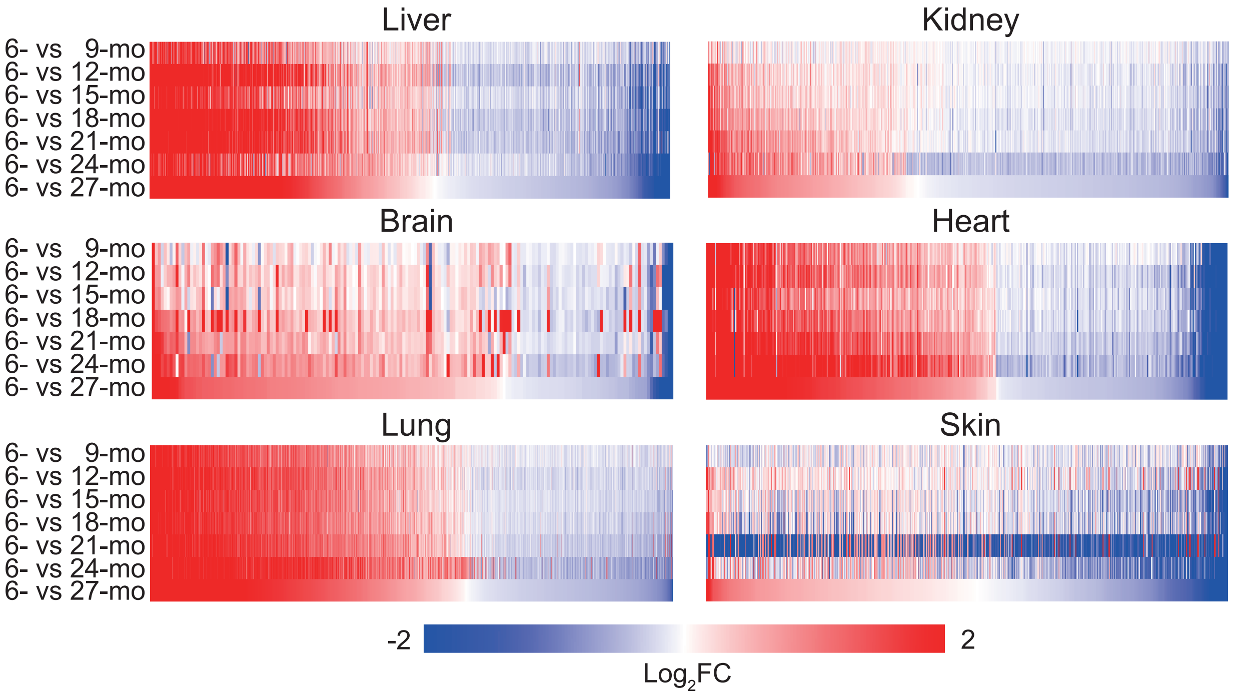


**Figure S5. Characterizations of age-related changes in gene expression in mouse tissues.** The heatmap shows $\log_2$ fold change gene expression in the age group shown in the left side of the figure compared to the 6 months old group. Genes that show significant age-dependent expression changes according to *Tabula Muris Senis* bulk RNA-Seq data are shown for mouse liver, kidney, brain, heart, lung, and skin.

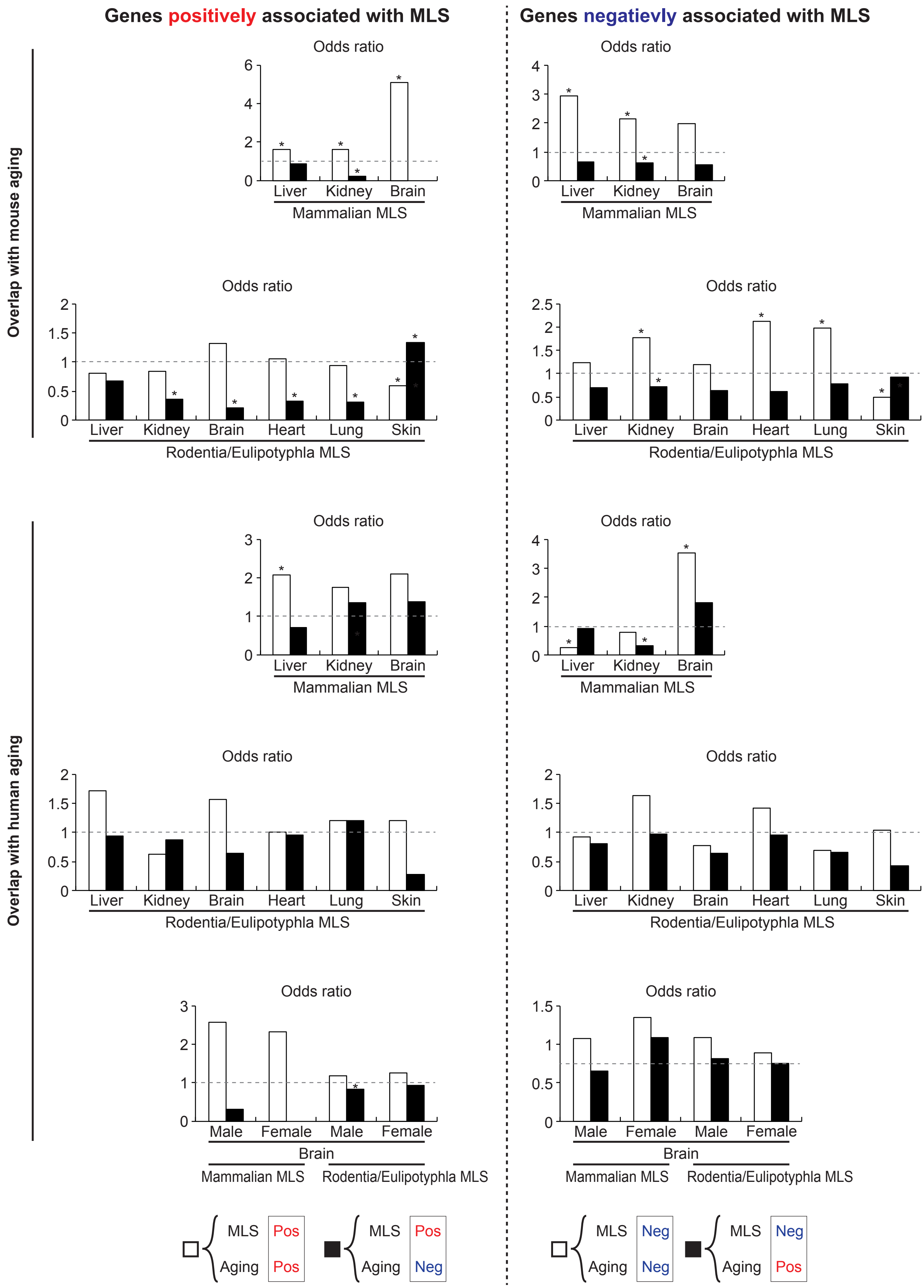


**Figure S6. Genes positively and negatively correlated with species MLS both contribute to the similarity between MLS- and aging-associated gene expression patterns.** The bar graphs

on the left side of the figure show the odds ratios of genes positively correlated with both species MLS and aging (white bars), and those of genes positively correlated with species MLS and simultaneously negatively correlated with aging (black bars). The bar graphs on the right side of the figure show the odds ratios of genes negatively correlated with both species MLS and aging (white bars), and those of genes negatively correlated with species MLS and simultaneously positively correlated with aging (black bars). * FDR < 0.05 (binominal test).

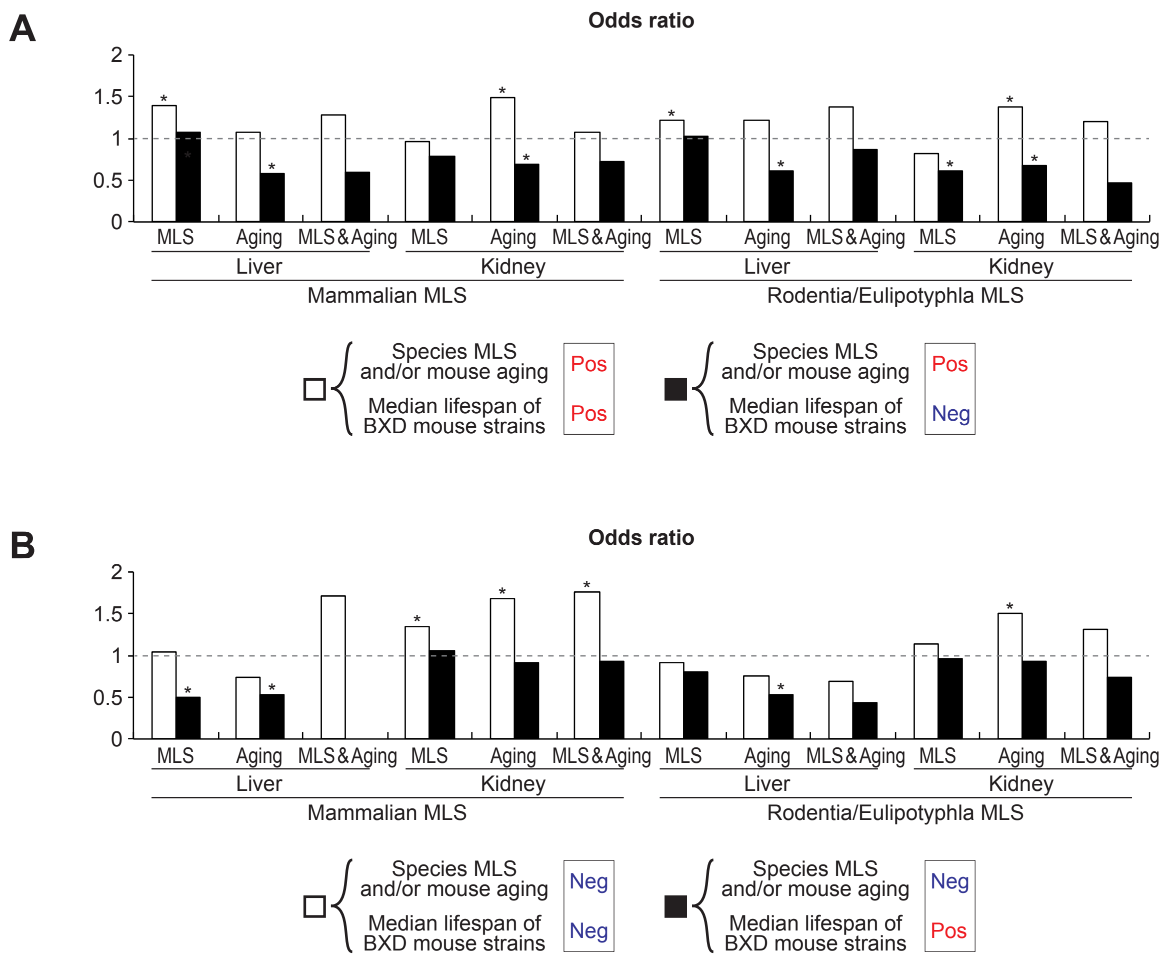


**Figure S7. Genes positively and negatively correlated with species MLS/mouse aging both contribute to the similarity between gene expression patterns associated with the lifespan of genetically heterogeneous mouse strains and those associated with species MLS/mouse aging.** (**A**) The white bars indicated as "MLS" show the odds ratios of genes positively correlated with both mouse lifespan and species MLS. The white bars indicated as "Aging" show the odds ratios of genes positively correlated with both mouse lifespan and mouse aging. The white bars indicated as "MLS & Aging" show the odds ratios of genes positively correlated with all mouse lifespan, species MLS, and mouse aging. The black bars indicated as "MLS" show the odds ratios of genes negatively correlated with mouse lifespan and simultaneously positively correlated with species MLS. The black bars indicated as "Aging" show the odds ratios of genes negatively correlated with mouse lifespan and simultaneously positively correlated with mouse aging. The black bars indicated as "MLS & Aging" show the odds ratios of genes negatively correlated with mouse lifespan and simultaneously positively correlated with both species MLS and mouse aging. (**B**) The white bars indicated as "MLS" show the odds ratios of genes negatively correlated with both mouse lifespan and species MLS. The white bars indicated as "Aging" show the odds ratios of genes negatively correlated with both mouse lifespan and mouse aging. The white bars indicated as "MLS & Aging" show the odds ratios of genes negatively correlated with all mouse lifespan, species MLS, and mouse aging. The black bars indicated as "MLS" show the odds ratios of genes positively correlated with mouse lifespan and simultaneously negatively correlated with species MLS. The black bars indicated as "Aging" show the odds ratios of genes positively correlated with mouse lifespan and simultaneously

negatively correlated with mouse aging. The black bars indicated as “MLS & Aging” show the odds ratios of genes positively correlated with mouse lifespan and simultaneously negatively correlated with both species MLS and mouse aging. * FDR < 0.05 (binominal test).
