# Supplementary material for: Gene expressions associated with longer lifespan and aging exhibit similarity in mammals
Source: Nucleic Acids Res. 2023 Jun 23;51(14):7205–19. doi: 10.1093/nar/gkad544 (PMC10415134; doi:10.1093/nar/gkad544)
Supplement: gkad544_Supplemental_Files [file gkad544_supplemental_files.zip › Supplementary_Table_Legends.docx]

**Supplementary Table S1**

Information of mammalian species, animals, and their RNA-Seq data used in the current study.

**Supplementary Table S2**

Numbers of MLS-associated genes, aging-associated genes, and their overlap.

**Supplementary Table S3**

Lists of mammalian MLS-associated genes.

**Supplementary Table S4**

Genes correlated with mouse aging, mammalian MLS, body weight, or age of sexual maturity.

**Supplementary Table S5**

Genes correlated with mouse aging or Rodentia/Eulipotyphla MLS.

**Supplementary Table S6**

Genes correlated with human aging or species MLS

**Supplementary Table S7**

Average number of transcription factor binding motifs per promoter in mammalian MLS-associated genes.
